# Implant-based reconstruction and adjuvant radiotherapy in breast cancer patients—current status and DEGRO recommendations

**DOI:** 10.1007/s00066-024-02334-3

**Published:** 2025-01-09

**Authors:** M. D. Piroth, D. Krug, R. Baumann, V. Strnad, K. Borm, S. Combs, S. Corradini, M. N. Duma, J. Dunst, G. Fastner, P. Feyer, R. Fietkau, W. Harms, T. Hehr, J. Hörner-Rieber, C. Matuschek, C. Schmeel, W. Budach, Breast Cancer Expert Panel of the German Society of Radiation Oncology (DEGRO)

**Affiliations:** 1 Department of Radiation Oncology, HELIOS University Hospital Wuppertal, Witten/Herdecke University, Heusnerstraße 40, 42283 Wuppertal, Germany; 2 Department of Radiotherapy and Radiation Oncology, University Medical Center Hamburg-Eppendorf, Hamburg, Germany; 3 Department of Radiation Oncology, St. Marien-Krankenhaus, Siegen, Germany; 4 Department of Radiation Oncology, University Hospital Erlangen, Erlangen, Germany; 5 TUM School of Medicine, Department of Radiation Oncology, Technical University of Munich, Munich, Germany; 6 Partner Site Munich, Deutsches Konsortium für Translationale Krebsforschung (DKTK), Munich, Germany; 7 Department of Radiation Medicine (IRM), Helmholtz Zentrum München (HMGU), Neuherberg, Germany; 8 Department of Radiation Oncology, University Hospital, LMU Munich, Munich, Germany; 9 Department of Radiation Oncology, Helios Clinics of Schwerin-University Campus of MSH Medical School Hamburg, Schwerin, Germany; 10 Department for Human Medicine, MSH Medical School Hamburg, Hamburg, Germany; 11 Department of Radiation Oncology, University Hospital Schleswig-Holstein, Kiel, Germany; 12 Department of Radiotherapy and Radio-Oncology, University Hospital Salzburg, Landeskrankenhaus, Paracelsus Medical University, Salzburg, Austria; 13 Formerly Department of Radiation Oncology, Vivantes Hospital Neukölln, Berlin, Germany; 14 Department of Radiation Oncology, St. Claraspital, Basel, Switzerland; 15 Department of Radiation Oncology, Marienhospital Stuttgart, Stuttgart, Germany; 16 Department of Radiation Oncology, University Hospital Düsseldorf, Düsseldorf, Germany; 17 Department of Radiotherapy and Radiation Oncology, Bielefeld University, Medical School and University Medical Center OWL, Campus Klinikum Mitte, Bielefeld, Germany; 18 Department of Radiation Oncology, University Hospital Bonn, Bonn, Germany

**Keywords:** Breast cancer, Breast reconstruction, Radiotherapy, Postmastectomy radiotherapy, Implant-based reconstruction, Mastectomy

## Abstract

**Purpose:**

The aim of this review is to give an overview of the results of prospective and retrospective studies using allogenic reconstruction and postmastectomy radiotherapy (PMRT) in breast cancer and to make recommendations regarding this interdisciplinary approach.

**Materials and methods:**

A PubMed search was conducted to extract relevant articles from 2000 to 2024. The search was performed using the following terms: (breast cancer) AND (reconstruction OR implant OR expander) AND (radiotherapy OR radiation). Data from the literature on allogenic breast reconstruction and radiation are presented and discussed in relation to toxicity and cosmesis.

**Conclusion and recommendations:**

Breast reconstruction is also feasible if PMRT is necessary. Patients need to be informed about the relevant risk of capsular fibrosis and implant failure. A planned reconstruction is no reason to forgo PMRT nor is an indication for PMRT a reason to forego implant-based breast reconstruction if desired by the patient. It is important to provide detailed information here to enable shared decision-making. There is still no clear consensus regarding implant-based reconstruction (IBR) and PMRT. However, in clinical practice, both a one-stage (immediate “implant-direct” IBR) procedure with PMRT up to the final implant and a two-stage (immediate-delayed IBR) procedure with PMRT up to the tissue expander (TE) and later exchange of the TE are used; both approaches have their specific advantages and disadvantages. Depending on patient-specific factors and the surgeon’s experience and estimates, both IBR procedures are also possible in combination with PMRT. When using a TE/implant approach, completing skin stretching by adequately filling the expander before PMRT may be favorable. This approach is particularly practical when adjuvant chemotherapy is planned but may lead to postponement of radiotherapy when primary systemic therapy is given. According to the latest data, moderate hypofractionation also appears to be safe in the context of the IBR approach. It is important to have a closely coordinated interdisciplinary approach and to fully inform patients about the increased rate of potential side effects.

## Introduction

*“The implications of performing postmastectomy reconstruction in the setting of radiotherapy, however, are both profound and controversial ….”* This statement expressed by Reavey and Mc Carthy in 2008 is still topical [1].

Breast-conserving therapy (BCT) is possible in 70–80% of cases as part of the primary treatment of breast cancer (BC) [2, 3]. Nevertheless, in up to 30% of cases, mastectomy is still unavoidable for maintaining oncological safety. However, from a psychological point of view, mastectomy is commonly perceived as very traumatic and has a considerable impact on patients’ quality of life [4]. At this point, reconstructive breast surgery offers the possibility of reconstituting the body image to reduce the psychological trauma. However, in the case of relevant risk factors, postmastectomy radiotherapy (PMRT) is indicated to improve locoregional control and reduce breast cancer mortality [5–7].

However, PMRT can impair the cosmetic outcome after reconstructive surgery with either allogenic implants (expander/silicone implants) or autologous tissue. In order to achieve the optimal cosmetic result and to keep potential late side effects as low as possible, a very good coordination between surgery and radiotherapy is mandatory. So far, the optimal procedure and timing of breast reconstruction and PMRT are still controversial and not well established [8].

In principle, two reconstructive procedures are available: implant-based reconstruction (IBR) and autologous reconstruction (AR). Each procedure has advantages and disadvantages, and no one procedure is appropriate for all patients. Factors that must be considered in the decision-making process include the localization and extent of the tumor, the extent of resection, patient-related medical and surgical risk factors, sufficient availability of local and/or distant autologous tissue, and especially the patient’s preference [1, 9–11].

Knowledge of the various options for reconstruction as well as of the risks and possible complications is the key to achieving a satisfactory result and, above all, enabling the patient to take part in shared decision-making.

Capsular fibrosis is one of the most common complications after IBR and one of the most frequent reasons for revision surgery [12]. Capsular contracture may have a considerable influence on the patient’s subjective and objective reconstruction results and must be assessed in the long term.

The reconstruction procedures are measured according to various items, in particular the more subjective items such as patient satisfaction and surgeon satisfaction and the more objective items such as the occurrence of complications like capsular fibrosis or complete reconstruction failure.

The aim of the present work is to give an overview of acute and chronic toxicity as well as of the cosmetic results of patients who received PMRT after IBR and to make recommendations regarding this interdisciplinary approach.

Data from the literature on breast reconstruction and PMRT are presented and discussed in relation to toxicity and cosmesis.

## Methods

A PubMed search was conducted to extract relevant articles from 2000 to 2024. The search was performed using the following terms: (breast cancer) AND (reconstruction OR implant OR expander) AND (radiotherapy OR radiation).

The aim of this study is to give an overview of the results of both prospective and retrospective studies using IBR and PMRT in breast cancer and to summarize further treatment aspects.

## Results

### Key measurement instruments and definitions in the literature relating to the reconstructive outcome

An established tool to assess and quantify the result of IBR based on clinical (i.e., firmness of tissue) and visual factors is the classification by Spear and Baker [13]. This classification divides the changes into class IA (reconstructed breast appears absolutely natural), IB (soft but visible implant), II (implant with mild firmness), III (implant with moderate firmness), and IV (excessively firm and symptomatic breast). If the implant has to be removed due to complications, this is called reconstruction failure. In addition, the BREAST‑Q questionnaire is widely used to assess patient-reported outcomes in a systematic and structured manner [14].

### Implant-based versus autologous reconstruction

In general, as shown recently by Broyles et al. when performing a meta-analysis by screening over 15,000 citations and including 40 studies, AR leads to better sexual wellbeing and satisfaction with breasts and also to a lower risk of reconstruction failure compared to IBR [10]. Just recently, the significantly higher satisfaction of patients with their breast and quality of life after AR versus IBR was confirmed by Nelson et al. when analyzing the outcomes following postmastectomy breast reconstruction based on an 8‑year examination of more than 3000 patients [15]. However, the study by Nelson et al. also showed very stable results regarding satisfaction and quality of life after IBR, illustrated in the BREAST‑Q [14] scores of 71.92, 75.63, and 76.98 (range 0–100) for “satisfaction with outcome,” “psychosocial wellbeing,” and “physical wellbeing with chest” after 5 years [15]. Disadvantages of AR include the longer duration of the operation and the more prolonged recovery without donor-site morbidity and a higher risk of thromboembolism [16, 17]. The need to establish a multidisciplinary approach with plastic/reconstructive surgery may be a further impediment regarding widespread use of AR in clinical practice.

### Adjuvant PMRT as a risk factor for increased side effects

Adjuvant therapy, in particular PMRT, is an important factor to consider regarding the outcome of breast reconstruction. Complication rates are much higher in irradiated than in non-irradiated patients who have received reconstructive surgery [18–21]. In a retrospective analysis including 280 patients (59 patients with RT, 221 patients without RT) with IBR, Sun et al. showed an overall complication rate of 50.8% vs. 33% in irradiated vs. non-irradiated patients, respectively [18]. In detail, significantly more reconstructive failures (18.6% vs. 8.6%), upper limb edema (10.2% vs. 2.3%), wound dehiscence (15.3% vs. 6.8%), and infections (22.0% vs. 9.5%) were seen.

In general, the type of reconstruction, in particular AR vs. IBR, is very determining for radiation-induced side effects. If PMRT is necessary, a two-stage procedure with autologous tissue (mastectomy → PMRT → AR) is considered the best approach by some authors [9, 20, 22–26]. Commonly, a time window of at least 6 months between radiotherapy and AR is considered appropriate [9]. However, the data are limited with regard to the best time interval. In a study by Momoh et al., no significant differences in complications were observed if PMRT was carried out at earlier than 6 months [27]. In a recent cross-sectional survey of 477 experienced plastic surgeons, 44.5% and 34.6% favored a time interval of 4–6 and 7–12 months between PMRT and AR, respectively [28].

In contrast to the sequence mentioned above (mastectomy → PMRT → AR), there are numerous analyses that have shown that immediate AR followed by PMRT shows no significant increase in complications or late side effects and results in high patient satisfaction [22, 29–31]. Jagsi et al. performed a prospective multicenter cohort study analyzing the impact of PMRT on complications and patient-reported outcomes after breast reconstruction [32]. The authors included 622 irradiated and 1625 non-irradiated patients. In patients receiving AR, PMRT did not lead to an impairment in patient-reported satisfaction or to an increase in complications. Accordingly, 30–40% of the participants of the 2021 consensus conference of the Oncoplastic Breast Consortium with live voting considered immediate AR to be the preferred method in the case of planned AR and expected PMRT. Delayed-immediate or delayed AR with or without an expander was considered a preference by 36–48% and 7–18%, respectively [33].

Further aspects regarding the relationship between autologous reconstruction and radiation, among other things timing, will be presented in a currently planned further paper by the breast cancer expert panel of the German Society of Radiation Oncology (DEGRO).

### Implant-based reconstruction and PMRT

Despite the advantages described above, AR is performed less frequently, which may be related to higher patient-related and medical costs and, ultimately, to financial challenges. In practice, the most common technique for reconstruction is IBR, which is performed 70% [34] up to 90% [15] of cases. The indication regarding the type of reconstruction depends, among other factors, on whether adjuvant radiotherapy is planned or not. The question is, what kind of factors play a role in achieving the optimal reconstructive outcome of IBR followed by PMRT?

### Timing of reconstructive and therapeutic procedures

The timing of the surgical procedure with IBR and radiation may have a significant impact on the reconstructive outcome. However, the results and recommendations regarding the optimal timing are not consistent. In this context, it should be noted that different implant-based approaches and nomenclatures are used in the literature to indicate the type and timing of the reconstruction and PMRT:

- *“Immediate” IBR and PMRT*: In this procedure the implant is inserted immediately, i.e., during the same surgical session as mastectomy. PMRT is carried out following immediate IBR.
- *“Delayed,” “delayed-immediate,” “immediate-delayed,” or “immediate two-stage” IBR and PMRT*: Delayed IBR, delayed-immediate IBR, or immediate two-stage IBR are different terms used by authors or working groups for a similar procedure meaning that a tissue expander (TE) is placed during mastectomy, which is followed by PMRT and then replacement by the final implant at a time interval after finishing PMRT.

### Outcomes depending on the different IBR procedures in detail

In general, clinical practice is highly heterogenous regarding timing and IBR procedures. Table 1 gives an overview over the current literature, which will be discussed in the following paragraphs.

#### Immediate IBRT

**Table 1:** Summary of trials on postmastectomy radiotherapy and implant-based reconstruction (IBR)

First author, year	Implant-based reconstruction	Design	Approach	Subgroup	Patients (*n*)	Median age (years)	Follow-up (months)	Capsular fibrosis grade ≥ III (%)	Reconstructive failure (%)	BREAST‑Q satisfaction breast	Wound dehiscence (%)	Infection (%)	Aesthetic results	Major complications
Cordeiro, 2015 [42]	Delayed-intermediate	Cohort trial non-randomized	Non PMRT	No PMRT	1486	47.8	45.6	4.1	4.6	64.1	–	–	Very good to excellent 73.8%, poor to fair 4.6%	–
			TE–PMRT–implant	PMRT	94	46.1	30.1	17.12	18.1	57.2	–	–	Very good to excellent 44.5%, poor to fair 11.1%	–
			TE–implant–PMRT	PMRT	210	46.3	40.3	50.9	12.4	56.2	–	–	Very good to excellent 45.2%, poor to fair 17.1%	–
Nelson, 2022 [44]	Delayed-intermediate	Retrospective	No PMRT	No PMRT	2175	49.5	36	7.4	17.7	65.5	0.5%	–	–	–
			PMRT–TE–implant	PMRT	239	53.6	36	14.6	17.7	62.4	1.3%	–	–	–
			TE–PMRT–implant	PMRT	290	48.6	36	25.5	23.1	57.6	0.3%	–	–	–
			TE–implant–PMRT	PMRT	228	47.3	36	35.1	17.1	56.7	1.3%	–	–	–
Piroth, 2009 [41]	Delayed-intermediate	Retrospective	TE–PMRT–implant	PMRT	33	49	24.9	24.2	22.7	–	–	–	Very good to excellent 50%, poor to fair 18.2%	–
Cagli, 2022 [21]	Delayed-intermediate	Retrospective	No PMRT	No PMRT	78	50.3	69	4.0	3.9	–	–	3.9%	–	–
			TE + PMRT–implant	PMRT	55	49.4	55	35.7	36.4	–	–	7.3%	–	–
	Immediate reconstruction		Implant–PMRT	PMRT	50	51.08	84	60.0	14.0	–	–	10.0%	–	–
Nava, 2011 [45]	Mix implant	Prospective cohort	No PMRT	No PMRT	98	49	50	24.1	2.3	–	–	–	Patients´opinion: good 68.1%, bad 2.1%	–
			TE + PMRT–implant	PMRT	50	49	50	53.3	40.0	–	–	–	Patients´opinion: good 46.2%, bad 7.6%	–
	Immediate reconstruction		Implant–PMRT	PMRT	109	49	50	57.8	6.4	–	–	–	Patients´opinion: good 52.2%, bad 11.1%	–
Sun, 2022 [18]	Delayed-intermediated and immediate	Retrospective	No PMRT	No PMRT	216	40.54	51.3	–	8.6	–	6.8%	9.5%	–	–
			TE + PMRT–implant or implant–PMRT	PMRT	59	36.71	37.2	–	18.6	–	15.3%	22.0%	–	–
Naoum, 2020 [38]	Immediate reconstruction	Retrospective	Implant–XRT	PMRT	171	49.7	43.3	7.0 (“require the release of capsule”)	18.1	–	–	6.4%	–	–
			Implant	No PMRT	245	52.4	49.9	1.1	12.4	–	–	2.6%	–	–
	Immediate-delayed		TE–PMRT–implant	PMRT	236	46.5	75	15.3 (“require the release of capsule”)	38.7	–	–	15.7%	–	–
			TE–implant	No PMRT	323	49.1	73	4.6	21.2	–	–	5.8%	–	–
Jagsi, 2018 [32]	Immediate reconstruction	Prospective multicenter cohort study	No PMRT	No PMRT (1 year^a^)	1218	50	12	–	3.5	65	1.0%	7.9%	–	Major Complications: 14.9%
				No PMRT (2 years^a^)	964	50	24	–	3.7	65.4	1.0%	9.3%	–	Major Complications: 15.6%
			Mixed implant procedure–PMRT	PMRT (1 year^a^)	386	50	12	–	12.2	55.6	2.8%	14.3%	–	Major Complications: 25.7%
				PMRT (2 years^a^)	283	50	24	–	18.7	54.2	7.4%	18.4%	–	Major Complications: 33.2%
	Autologous reconstruction		No PMRT	No PMRT (1 year^a^)	407	50	12	–	2.5	68.5	2.0%	3.4%	–	Major Complications: 24.1%
				No PMRT (2 years^a^)	332	50	24	–	2.4	69	2.4%	3.3%	–	Major Complications: 22.9%
			Autologous–PMRT	PMRT (1 year^a^)	236	50	12	–	0.4	63.9	5.1%	5.9%	–	Major Complications: 14.8%
				PMRT (2 years^a^)	199	50	24	–	1.0	65.1	5.5%	4.5%	–	Major Complications:17.6%
Vinsensia, 2024 [55]	Immediate implant-based reconstruction	Retrospective	Implant–PMRT	PMRT	118	45	22	22.9	25.4	–	–	–	–	–
Boniface, 2022 [60]	Implant-based immediate	Population-based cohort	No PMRT	No PMRT	856	50	72	–	7.9	–	–	–	–	–
			Implant or expander–PMRT	PMRT	749	47	72	–	21.1	–	–	–	–	–
			PMRT–implant or expander	Previous RT	144	55	72	–	27.8	–	–	–	–	–

*PMRT* postmastectomy radiotherapy, *IBR* implant-based reconstruction, *TE* tissue expander, BREAST-*Q* Score to measure patient-reported outcome in breast surgery (score 1–100) [14]

^a^Follow-up

This approach is attractive because of avoiding a second operation and also the procedure of tissue expander (TE) expansion over weeks to months.

Skin-sparing mastectomy (SSM) and nipple-sparing mastectomy (NSM) are widely used to further improve cosmetic outcomes. Among others, these advancements in mastectomy techniques and increasing clinical experience have encouraged surgeons to more commonly perform an immediate implant-based reconstruction (IBR) approach with direct placement of the implant [35–37].

Naoum et al. retrospectively analyzed the outcome of 1814 patients, who had undergone immediate autologous or implant-based breast reconstruction or a two-stage approach using a TE and delayed autologous or implant-based breast reconstruction. PMRT was administered in approximately 40% of patients, regardless of the type of reconstruction. In the case of implementation of a TE, PMRT was given before final reconstruction in most patients [38].

In cases of PMRT, the overall implant failure rates were 29.7% and 16.4% for immediate-delayed IBR vs. immediate “direct-to-implant” IBR, respectively. Without PMRT the values were 14.5% and 8.6%. In the multivariable analysis, immediate-delayed IBR was associated with a significantly higher complication rate compared to immediate “direct-to-implant” IBR regarding infections (OR 2.9; *p* = 0.004), skin necrosis (OR 3.02; *p* = 0.018), capsular contracture (OR 2.63; *p* = 0.009), and overall implant failure (OR 3.09; *p* < 0.001). No significant difference was seen between immediate IBR and AR. Impressively, the authors could show that in cases of PMRT, immediate IBR complication rates were not significantly different from AR complication rates, and they state that the immediate “direct-to-implant” IBR approach may offer a valuable option for patients receiving PMRT [38].

#### Delayed, delayed-immediate, immediate-delayed, or immediate two-stage IBR and PMRT

If immediate IBR is not possible or desired, e.g., because skin expansion is necessary, the delayed-immediate IBR procedure is used in the allogeneic setting. According to Kronowitz et al., introducing this approach in the early 2000s, the TE was placed during mastectomy, followed by PMRT (during PMRT the expander was partly deflated) and then replaced by the final implant 3 months after finishing PMRT [26]. In this setting, a re-filling of the expander was carried out to scaffold the breast skin [26, 39]. The overall tissue expander loss rate was 32%. The reasons for expander loss were infection (53%), an irregular fold in the expander occurring during PMRT (27%), and skin flap necrosis (13%; as part of the implant-based reconstructive approach, use of autologous tissue was also permitted in some cases) [39].

Within a prospective multi-institutional study using a comparable two-stage approach, Berry et al. analyzed the factors associated with reconstruction failure in 141 consecutive patients. After a median follow-up time of 37 months, Baker III or IV capsular contracture was seen in 32.5%. Overall, 32 (22.7%) reconstruction failures occurred [40].

A reconstruction failure rate of 18.7% was seen in a study by Jagsi et al. using an implant-based approach followed by PMRT [32]. In a smaller study, using an approach adapted to Kronowitz [39] but filling the expander fully before starting PMRT, implant loss was observed in five out of 22 patients (22.7%). With regard to cosmesis, patients were very satisfied/satisfied in 50% of cases [41]. Further, 32.1% and 17.9% of patients were moderately satisfied and disappointed, respectively. Notably, 81% of patients would undergo reconstructive breast surgery again if mastectomy were necessary, 14.3% of patients were undecided, and 4.8% answered no.

Using the two-stage approach, Cordeiro et al. explored the question of the optimal timing of replacement of the expander with the implant (before or after PMRT) [42]. Overall, 1486 patients treated with expander-/implant-based reconstruction between 2003 and 2012 were included in the analysis. PMRT to the implant after replacing the TE (implant-RT) or to the TE (TE-RT) was performed in 210 and 94 patients, respectively; no PMRT was needed for 1143 patients. The follow-up times were 40.3, 30.1, and 45.6 months for the cohorts. Patients who received TE-RT had a higher rate of reconstruction failure (18.1%) than patients receiving implant-RT (12.4%) and no RT (4.6%). However, no significant difference was reached between TE-RT and implant-RT. Capsular contractures grade III/IV were seen in 17.1% (TE-RT), 50.9% (implant-RT), and 4.1% (no RT) of patients. The findings of lower rates of capsular contractures but a higher rate of implant loss with TE-RT were confirmed in a meta-analysis of 20 studies with 2348 patients [43].

According to Cordeiro et al., a lower rate of capsular contractures in the TE group can be explained by the fact that an aggressive capsulotomy was performed as part of the surgical exchange of the expander for the implant.

Further, the authors attribute the superior aesthetic outcome of TE-RT to the fact that the skin over the implant can be adapted or re-draped in the same surgical session as the one during which the expander is replaced after PMRT. A good and very good/excellent aesthetic outcome, as assessed by the surgeon, was seen in 44.4% and 44.5% (TE-RT) vs. 37.7% and 45.2% (implant-RT), respectively.

Recently, the same research group published an observational study with long-term follow-up [44]. The following cohorts were studied: no PMRT (*n* = 2175), TE-RT (*n* = 290), and implant-RT (*n* = 228). Further, a cohort with RT prior to mastectomy as part of previous breast-conservation therapy (*n* = 239) was included. The authors stated that patient-reported outcomes were not affected by the timing of radiotherapy. Nevertheless, capsular contracture was higher when performing implant-RT. Based on the findings, the authors concluded that the timing of radiotherapy does not appear to affect patients’ awareness regarding the reconstructive outcome. However, the higher capsular contracture rate following PMRT to the implant suggests performing PMRT before placement of the final implant.

Within the concept using PMRT after TE, the complete filling of the expander before PMRT to expand the skin completely may play an important role in the superior outcome. Nava et al. showed that the results are less favorable if radiotherapy is still given during the expander-filling phase. Compared to implant-RT, there was a higher rate of implant failure (40% vs. 6.4%) and also a higher incidence of Baker grade IV capsular fibrosis (13.3% vs. 10.1%) [45].

This approach is also supported by experimental studies by Dvali et al. and Goodman et al. [46, 47]. Goodman et al. used an animal model (New Zealand white rabbit) to examine the pathophysiological changes of the combination of tissue expansion and radiation therapy [46]. An expander was subcutaneously inserted around the animals’ backs. After consecutive filling of the expander, there was a waiting period of 2–3 weeks. This was followed by single-fraction irradiation with 20, 25, or 35 Gy to one half of the expanded skin. The other half was the internal control. The histological evaluation was performed 2 months later. The results showed an increasing thickening of the epidermis, depending on the radiation dose. However, the dermis was unaffected. Furthermore, there was no significant capsular fibrosis. The authors state that tissue expansion and subsequent stabilization over 2–3 weeks could lead to superior radiation therapy tolerance. Consistent with this interpretation, Dvali’s experimental studies reported that skin distensibility decreased significantly with prior irradiation [47].

However, the increasing use of neoadjuvant vs. adjuvant systemic therapy decreases the timespan available for adequate expander filling because adjuvant radiotherapy can often be started after a few weeks rather than after several months and may hence impair cosmetic outcome.

### Surgical aspects

#### Mastectomy technique

In the context of the reconstructive surgical procedure, several mastectomy techniques are available, including modified radical mastectomy (MRM), skin-sparing mastectomy (SSM), or nipple-sparing mastectomy (NSM). SSM and NSM have become increasingly accepted in recent years as standard operations in appropriately selected patients, as reports suggest no increased risk of disease recurrence compared to conservative mastectomies [48–53]. SSM was developed to improve cosmetic results for breast cancer patients, also allowing for immediate breast reconstruction by preservation of the skin envelope as well as the inframammary fold. In SSM, a large portion of skin with a rim of subcutaneous tissue is left in place. In NSM, also the whole nipple–areolar complex (NAC) is preserved to further improve cosmetic outcome. This usually requires an adequate distance from the tumor to the NAC and intraoperative assessment of the retroareolar margin.

With regard to capsular fibrosis rates, Hammond et al. found no significant differences between NSM (*n* = 262), SSM (*n* = 160), or simple mastectomy (*n* = 29), [54]. Also in an analysis by Vinsensia et al. could no significant differences in the rates of capsular contracture be seen with respect to the surgical techniques used (NSM, SSM, MRM) [55].

#### Pre- vs. subpectoral implantation

Regarding the issue of prepectoral or subpectoral implantation, prepectoral positioning appears to have advantages. In a very recent meta-analysis, which included 15 studies with over 3000 patients, lower rates of capsular contracture (odds ratio [OR] 0.54; *p* = 0.02) and implant failure (OR 0.58; *p* = 0.001) were found for the prepectoral approach [56]. The complication rates of seroma, hematoma, infection, and skin flap necrosis were not significantly different. There was also no significant difference in the BREAST‑Q scores [56].

In the case of PMRT, Sinnott et al. showed that the capsular contracture rates were three times higher in the subpectoral group vs. the prepectoral group (52.2% vs. 16.1%; *p* = 0.0018) [57]. The incidence of Baker grade III/IV contractures was 83.3% vs. 22.2% (*p* = 0.0092). In addition, in an analysis by Sobti et al., capsular contracture rates were lower performing prepectoral vs. subpectoral implants in irradiated patients [58].

For further details, we refer to a relevant paper written in collaboration with breast cancer surgeons from the European Breast Cancer Research Association of Surgical Trialists (EUBREAST), a breast pathologist from the Danish Breast Cancer Group (DBCG), and representatives from the European Society for Radiotherapy and Oncology (ESTRO) breast cancer course. The authors summarize the common types of mastectomies and reconstruction procedures with particular consideration of the challenges faced during surgery and later on target volume definition in the case of PMRT [59].

#### Postoperative complications

Vinsensia et al. found, based on a retrospective analysis (*n* = 118), that postoperative complications such as hematomas/seromas, prolonged wound healing, and pain and swelling in the surgical area have a significant influence on the rate of capsular contracture (HR 2.245; *p* = 0.011) [55]. Based on a population-based cohort study (*n* = 1749), Boniface et al. showed that two or more revision surgeries are an independent risk factor to increase the implant loss rate (HR 3.03; *p* < 0.001) [60].

### Target volume

ESTRO-ACROP (European Society for Radiotherapy and Oncology-Advisory Committee on Radiation Oncology Practice) has recently published a consensus guideline for target volume delineation in the setting of PMRT after IBR for early-stage breast cancer [61]. These recommendations are mainly based on anatomical considerations regarding the distribution of breast glandular tissue and regional lymphatic drainage. While conventionally the whole reconstructed breast including the implant or tissue expander were defined as the clinical target volume (CTV), the guideline panel developed a volume-based radiotherapy approach. The expert group aimed to reduce potential complications by tailoring the target volume to tissues at risk of recurrence [61]. They state that after mastectomy, the CTV includes the remaining subcutaneous glandular tissue and the subcutaneous lymphatic tissue. The major pectoral muscle represents the anatomical dorsal boundary. The ventral part between the skin and the implant, including the subcutaneous lymphatic tissue, should be included in the CTV. Any remaining mammary gland tissue should also be included. The recommendation differs between subpectoral and prepectoral placement of the implant. In the case of a subpectoral implant, the rim of tissue ventral to the major pectoral muscle and the implant comprises the CTV plus parts of the chest wall surrounding the pectoral muscle around which the lymphatics flow. Hence, the implant or tissue expander are largely excluded from the CTV according to the guideline. In addition, transposed tissue (skin, fatty tissue, muscle) is not part of the CTV. For patients with subpectoral implants, according to the ESTRO guideline, the volume posterior to the implant, between the implant and the pectoral muscle/chest wall, should only be included into the CTV if risk factors are given, such as a large primary tumor (pT3) or infiltration of the pectoral muscle/thoracic wall.

We believe that the abovementioned modifications to the CTV should be approached with caution, given the limited clinical data available in this context.

Historically and currently, a tangential technique is used in most cases, which, in addition to the prepectoral tissue and the implant/expander, also covers the region dorsal to the implant, including the fascia region and ventral parts of the pectoralis muscle.

This critical view is supported by an important commentary on the abovementioned guideline by R. W. Mutter from the Mayo Clinic [62]. Kaidar-Person et al. responded to this letter and used it as an opportunity to concretize and realign some aspects [63]. The authors emphasized that the guidelines recommend that for high-risk patients, such as those with locally advanced breast cancer (for example with residual disease after primary systemic therapy), treatment is to be individualized based on a multidisciplinary discussion. Further, they pointed out that in case of uncertainty, treatment should be performed in a manner similar to traditional tangential fields, including the implant and the retropectoral areas.

As also pointed out in an earlier report of our working group [64], the currently available data do not support reducing the target to the nipple–areola complex only (in case of a nipple-sparing mastectomy) or to the tissue only behind the expander. The evidence for performing an implant-sparing approach is very sparse with regard to possible advantages or even prognostic disadvantages in the case of omitting parts of the implant region [65]. Preliminary data using a HALFMOON (“helical altered fractionation for implant partial omission”) technique and target contouring according to the ESTRO-ACROP recommendations were presented at ESTRO 2023. After a median of 1.2 years, 28 of 32 patients for whom the information regarding capsular fibrosis was present showed capsular fibrosis Baker grade ≥ II [65]. These preliminary data suggest that an implant-sparing radiotherapy concept may not lead to a relevant improvement in the capsular contracture rate. Further prospective studies are necessary to analyze this question.

It should also be mentioned in this context that the only techniques with which implant sparing can be adequately realized, such as VMAT or helical tomotherapy, have other disadvantages, such as a higher integral dose and a potential increase in the doses to the contralateral breast, lungs. and heart.

Generally, preoperative evaluation of the planned procedure in patients who are expected to receive PMRT should be carried out by the breast surgeon as well as the radiation oncologist or be discussed together in the tumor board [61].

Ideally, modifications of the target volume should be tested in the setting of a prospective clinical trial or registry to demonstrate oncological safety as well as improvements in morbidity and quality of life. Based on the limited data and the abovementioned technical aspects, we consider including the complete tissue in front of and behind the implant into the CTV as standard of care for most cases. However, treatment with reduced volumes may be individually discussed based on the ESTRO recommendations [61].

### Fractionation

In the case of breast-conserving surgery, moderate hypofractionation is standard of care in adjuvant radiotherapy of the breast and is increasingly applied for radiotherapy of the chest wall after mastectomy [66]. This is supported by a prospective randomized study by Wang et al. In this phase III noninferiority study, 820 patients who had undergone mastectomy and axillary dissection and had ≥ 4 positive nodes or pT3–4 were included. Patients were randomized to receive chest wall and nodal irradiation at a dose of 50 Gy in 25 fractions or 43.5 Gy in 15 fractions. It could be shown that PMRT and regional irradiation using moderate hypofractionation was noninferior to conventionally fractionated radiotherapy in terms of locoregional control. Long-term toxicity was similar but acute toxicity favored moderate hypofractionation. However, breast reconstruction was not permitted in this trial. It should also be emphasized that the hypofractionated regimen used by Wang with 43.5/2.9 Gy applied a biologically higher dose than the generally recommended regimen of 40/2.67 Gy (EQD2 51.3 Gy vs. 45.5 Gy for alpha/beta = 3).

Recently, results from the randomized controlled FABREC trial were published by Wong et al. [67]. This study compared quality of life (QOL) and clinical outcomes of moderately hypofractionated (42.54 Gy/16 fractions) vs. conventionally fractionated (50 Gy/25 fractions) PMRT in the setting of immediate implant-based reconstruction. After a median follow-up of 31.8 months, the physical wellbeing and overall toxicity were comparable between the two arms for the 385 patients. The use of a tissue expander was associated with a significantly increased risk of chest wall toxicity (hazard ratio 4.44) in multivariable analysis. The authors conclude that their early results support the use of moderate hypofractionation in the setting of TE- or implant-based breast reconstruction. This is supported by findings from retrospective cohort studies [68–70].

Mutter et al. conducted a randomized controlled trial of conventionally fractionated vs. moderately hypofractionated proton PMRT [71]. Breast reconstruction was used in 70% of patients, with the majority having TE placement. At a median follow-up of 39.3 months, the rate of protocol-defined complications was similar between the arms; however, noninferiority could not be demonstrated. Limitations include the sample size of 82 patients and dosimetric uncertainties related to the use of proton radiotherapy in patients with TE.

In line with these recent data, 86.9% of the expert panel of ESTRO-ACROP voted to offer moderate hypofractionation regardless of breast reconstruction [72].

Just recently, the primary analysis of the randomized controlled RT CHARM trial was presented at ASTRO 2024 [73]. This trial conducted by the alliance group randomized 898 patients to conventional fractionation (50 Gy in 25 fractions) or moderate hypofractionation (42.56 Gy in 16 fractions), with stratification according to the timing (immediate vs. delayed) and type (autologous or implant-based) of reconstruction. The primary endpoint was the rate of reconstruction-associated complications after 2 years. Noninferiority of moderate hypofractionation was demonstrated. Autologous reconstruction (vs. implant-only reconstruction) was associated with a reduced risk of reconstruction-associated complications, whereas immediate-delayed reconstruction (as compared to immediate reconstruction) demonstrated a higher risk of complications.

### Neoadjuvant radiotherapy

If radiotherapy is indicated, its use before mastectomy and immediate reconstruction with autologous flaps may potentially have advantages. It reduces the risk of flap shrinkage and fibrosis because the flaps are not exposed to radiation. Matuschek et al. highlighted that 60% of patients in a monocentric cohort reported excellent or good long-term cosmetic results after preoperative radiotherapy and mastectomy with autologous reconstruction [74].

Furthermore, the PRADA study demonstrated that preoperative hypofractionated radiotherapy combined with skin-sparing mastectomy and DIEP flap reconstruction may be feasible and safe [75]. However, this trial only included 33 patients; a confirmatory trial is underway. Schaverien et al. recently published results of a randomized controlled phase II trial of preoperative radiotherapy in patients scheduled for immediate breast reconstruction [76]. Patients were randomized to receive 50 Gy in 25 fractions (45 Gy to the regional nodes) or 40.05 Gy in15 fractions (37.5 Gy to the regional nodes). Among 49 evaluable patients, 46 underwent autologous reconstruction. There were no flap losses, but 21% of patients had major postoperative complications and both patients with TE had infections, resulting in TE explantation in one patient. The trial was not adequately powered to compare outcomes between fractionation schedules. Overall, preoperative radiotherapy represents a promising approach to enhance aesthetic outcomes and reduce treatment time in breast cancer patients undergoing mastectomy and reconstruction, warranting further research in trials like the ongoing NeoRad study: ‘Preoperative radiotherapy versus postoperative radiotherapy after neoadjuvant chemotherapy (“NeoRad”) in high-risk breast cancer: a prospective, randomized, international multicenter phase III trial’ (NCT04261244). This study aims to compare the effectiveness and safety of preoperative versus postoperative radiotherapy in high-risk breast cancer patients irrespective of the type of breast surgery.

## Conclusion

Breast reconstruction is feasible if PMRT is necessary. Patients need to be informed about the associated risk of capsular fibrosis and implant failure. A planned reconstruction is not a reason to withhold PMRT nor is an indication for PMRT a reason to forego breast reconstruction if desired by the patient. It is important to provide detailed information in order to enable shared decision-making. There is still no clear consensus regarding IBR and PMRT. However, in clinical practice, both a one-stage (immediate “implant-direct” IBR) procedure with PMRT to the final implant and a two-stage (immediate-delayed IBRT) procedure with PMRT to the TE and later exchange of the TE are used; both approaches have their specific advantages and disadvantages.

Depending on patient-specific factors and the surgeon’s experience and estimates, both IBR procedures are also possible in combination with PMRT.

When using a TE/implant approach, completing skin stretching by adequately filling the expander before PMRT may be favorable. This approach is particularly practical when adjuvant chemotherapy is planned but it may lead to postponement of radiotherapy when primary systemic therapy is given.

According to the latest data, moderate hypofractionation also appears to be safe in the context of the IBR approach.

It is of the utmost importance to adopt a meticulous interdisciplinary approach and to ensure that patients are fully informed about the elevated risk of potential adverse effects.
